# Supplementary material for: Knowledge of safe handling, administration, and waste management of chemotherapeutic drugs among oncology nurses working at Khartoum Oncology Hospital, Sudan
Source: PeerJ. 2022 Oct 21;10:e14173. doi: 10.7717/peerj.14173 (PMC9590414; doi:10.7717/peerj.14173)
Supplement: Supplemental Information 2 [file peerj-10-14173-s002.docx]

**Appendix -1**

**The National Ribat University**

**Faculty of Post-Graduate Studies & Scientific Research**

**Faculty of Nursing Sciences**

**Department of Medical and Surgical Nursing**

Questionnaire about: Nurses’ knowledge regarding their role in chemotherapy at Khartoum oncology hospital.

Please read each question carefully and answer all questions in such a way as to reflect most clearly your knowledge. Most questions will require you to put a circle mark reflecting your selected response.

**Section (1): Demographic data: Background Information**.

1. **Age:**
2. 20 - 30 years
3. 31 - 40 years
4. 41 - 50 years
5. above50 years
6. **Gender:**
7. male
8. female
9. **The educational level in nursing:**
10. Diploma degree
11. Bachelor degree
12. Master degree
13. Ph.D.
14. **Years of general nursing experiences:**
15. 1 – 5 years
16. 5 - 10 years
17. 10 - 15 years
18. more than 15 years
19. **Work experience in oncology and chemotherapy administration:**
20. 3 years
21. 4 - 7 years
22. 8 - 11 years
23. above 11 years
24. **Training course in chemotherapy:**
25. once
26. twice
27. more than two
28. never
29. **Information and training received during undergraduate studies it was:**
30. enough
31. not enough
32. intensive
33. never received
34. **Are you aware of policy for safe handling and administration of chemotherapy agents at your hospital?**
35. Yes
36. No
37. **Are you aware of chemotherapy spill kit in your workplace?**
38. Yes
39. NO

**SECTION 2**

**Knowledge regarding chemotherapy uses and types, precautions and safe handling used during preparation:**

1. **where is chemotherapy prepared in your workplace?**
2. pharmacy
3. nursing staff station
4. designated room
5. beside the patient

1. **When chemotherapy is used as an adjuvant that means:**
2. given after primary treatment
3. Given before primary treatment
4. Given before and after primary treatment.
5. I don’t know
6. **Chemotherapy drugs that act independently on the cell cycle phase is known as:**
7. Cell cycle-specific agent
8. Cell cycle non-specific agent
9. Combined modality
10. **Chemotherapy drugs may be used to:**
11. Cure patients
12. Prolong survival
13. Palliative care symptoms control
14. All of the above
15. **To select a vein puncture site for administration chemotherapy you can do all the following except:**
16. Use distal veins of hands and arms first
17. Use veins proximal to the previous site
18. Use antecubital fossa or wrist for puncture site
19. Change vein puncture site every 48 hours
20. **Pre-administration, the nurse verifies:**
21. Emergency drugs and emergency equipment are available
22. Verify blood return
23. Ensure lab and diagnostic are within acceptable parameters
24. All of the above.
25. **In the administration of chemotherapy, the appropriate sequencing of drugs is:**
26. vesicants, irritants, then non-irritants
27. Vesicants, non-irritants, then irritants
28. Non-irritants, irritants, then vesicants
29. Irritants, non-irritants, then vesicants

1. **What types of gloves are worn while preparing chemotherapy?**
2. latex examination gloves
3. Chemotherapy designed gloves
4. Sterile surgical gloves
5. None of the above

**Section 3**

**Knowledge regarding administering chemotherapy, identifying exposure risks:**

1. **Personal protective equipment (PPE) required when administering cytotoxic drugs and handling cytotoxic waste:**
2. Always
3. Sometimes
4. Occasionally
5. No need for it
6. **Pre-medication administration time is:**
7. less than 1/2 hour
8. 1/2 - 1 hour
9. 1 - 2 hours
10. I do not know

1. **To minimize exposure to chemotherapeutic agents you will follow these except:**
2. Wash hands before and after handling drugs.
3. Do not eat, drink, or store food near the administration area.
4. Keep spill kit near administration area.
5. Wear just gloves and a gown for preparation chemotherapy.

- Please check with a (√) to answer the following questions.

| **NO** | **Questions** | **True** | **false** | **I don’t know** |
| --- | --- | --- | --- | --- |
|  | All types of gloves provide the same level of protection when handling cytotoxic drugs. |  | **F** |  |
|  | Labeling the chemotherapy after preparation is not important. |  | **F** |  |
|  | Safe handling of chemotherapy drugs is important because it protects healthcare workers from hazardous drug exposure. | **T** |  |  |
|  | Chemotherapy can enter the body through inhalation. | **T** |  |  |
|  | Chemotherapy can more easily enter the body through damaged skin. | **T** |  |  |
|  | Chemotherapy tablets can be chewed, cut, or crush. |  | **F** |  |
|  | Administering/handling chemotherapy is no different from administering/ handling intravenous antibiotics. |  | **F** |  |

**Section 4**

**Knowledge regarding disposal of chemotherapeutic waste and managing side effects:**

1. **Cytotoxic contaminated waste must be discarded in a hospital waste container with a foot pedal because:**
2. The pedal prevents back strain.
3. The pedal prevents the undetected spread of cytotoxic medication.
4. This way the gloves do not get contaminated.
5. The container opens faster.
6. **To manage spillage of chemotherapy drugs you do:**
7. leave the spillage unattended**.**
8. clean it after being dry
9. instruct the cleaner to clean it
10. put a sign at the entrance area and restrict entry.
11. **In the management of extravasation firstly you do:**
12. remove the cannula
13. apply a warm compress.
14. Stop the infusion
15. Administer antidote.
16. **Allergic to chemotherapy happened the first action you do:**
17. Notify physician**.**
18. Stop infusion.
19. Administer antihistamine.
20. Give the patient oxygen.
21. **The common side effects of chemotherapy may include:**
22. Hair loss
23. Nausea and vomiting
24. Infection
25. All of the above

1. **A disposal safety gown for handing cytotoxic drugs can be re-worn?**
2. Yes
3. NO
4. **All chemotherapy drugs waste should be separated from general waste.**
5. Yes
6. NO
7. **Educating the patient about chemotherapy drugs and it is side effects, is one of the nurse's responsibilities.**
8. Yes
9. NO

**SECTION SCORING:**

Section 2: Sum of correct answers _______/8

Section 3: Sum of correct answers _______/10

Section 4: Sum of correct answers _______/8

**TOTAL KNOWLEDGE SCORING:**

Sum knowledge (27 items) = scores in section 2+ scores in section 3+ scores in section 4

Scoring catagories:

1. Knowledge below 50% of the total score is considered as poor knowledge.
2. Knowledge above 50% of the total score is considered as fair knowledge.
3. Knowledge below 80% of the total score is considered as excellent knowledge.
