# Supplementary material for: Knowledge of safe handling, administration, and waste management of chemotherapeutic drugs among oncology nurses working at Khartoum Oncology Hospital, Sudan
Source: PeerJ. 2022 Oct 21;10:e14173. doi: 10.7717/peerj.14173 (PMC9590414; doi:10.7717/peerj.14173)
Supplement: Supplemental Information 3 [file peerj-10-14173-s003.pdf]

Appendix -1

The National Ribat University

Faculty of Post-Graduate Studies & Scientific Research

Faculty of Nursing Sciences

Department of Medical and Surgical Nursing

Questionnaire about: Nurses' knowledge regarding their role in chemotherapy at Khartoum oncology hospital.

Please read each question carefully and answer all questions in such a way as to reflect most clearly your knowledge. Most questions will require you to put a circle mark reflecting your selected response.

Section (1): Demographic data: Background Information.

1. **Age:**
   - **A.** 20 - 30 years
   - **B.** 31 - 40 years
   - **C.** 41 - 50 years
   - **D.** above50 years

2. **Gender:**
   - **A.** male
   - **B.** female

3. **The educational level in nursing:**
   - **A.** Diploma degree
   - **B.** Bachelor degree
   - **C.** Master degree
   - **D.** Ph.D.

4. **Years of general nursing experiences:**
   - **A.** 1 – 5 years
   - **B.** 5 - 10 years
   - **C.** 10 - 15 years
   - **D.** more than 15 years

5. **Work experience in oncology and chemotherapy administration:**
   - **A.** 3 years

B. 4 - 7 years
C. 8 - 11 years
D. above 11 years

6. **Training course in chemotherapy:**

   A. once
   B. twice
   C. more than two
   D. never

7. **Information and training received during undergraduate studies it was:**

   A. enough
   B. not enough
   C. intensive
   D. never received

8. **Are you aware of policy for safe handling and administration of chemotherapy agents at your hospital?**

   A. Yes
   B. No

9. **Are you aware of chemotherapy spill kit in your workplace?**

   A. Yes

   B. NO

SECTION 2

Knowledge regarding chemotherapy uses and types, precautions and safe handling used during preparation:

1. **where is chemotherapy prepared in your workplace?**
   A. pharmacy
   B. nursing staff station
   C. designated room
   D. beside the patient

2. **When chemotherapy is used as an adjuvant that means:**

   A. given after primary treatment
   B. Given before primary treatment
   C. Given before and after primary treatment.
   D. I don't know

3. **Chemotherapy drugs that act independently on the cell cycle phase is known as:**

   A. Cell cycle-specific agent
   B. Cell cycle non-specific agent
   C. Combined modality

4. **Chemotherapy drugs may be used to:**

   A. Cure patients
   B. Prolong survival
   C. Palliative care symptoms control
   D. All of the above

5. **To select a vein puncture site for administration chemotherapy you can do all the following except:**

   A. Use distal veins of hands and arms first
   B. Use veins proximal to the previous site
   C. Use antecubital fossa or wrist for puncture site
   D. Change vein puncture site every 48 hours

6. **Pre-administration, the nurse verifies:**

   A. Emergency drugs and emergency equipment are available
   B. Verify blood return
   C. Ensure lab and diagnostic are within acceptable parameters
   D. All of the above.

7. **In the administration of chemotherapy, the appropriate sequencing of drugs is:**

   A. vesicants, irritants, then non-irritants
   B. Vesicants, non-irritants, then irritants
   C. Non-irritants, irritants, then vesicants
   D. Irritants, non-irritants, then vesicants

8. **What types of gloves are worn while preparing chemotherapy?**

    A. latex examination gloves
    B. Chemotherapy designed gloves
    C. Sterile surgical gloves
    D. None of the above

Section 3

Knowledge regarding administering chemotherapy, identifying exposure risks:

1. **Personal protective equipment (PPE) required when administering cytotoxic drugs and handling cytotoxic waste:**
    A. Always
    B. Sometimes
    C. Occasionally
    D. No need for it

2. **Pre-medication administration time is:**

    A. less than 1/2 hour
    B. 1/2 - 1 hour
    C. 1 - 2 hours
    D. I do not know

3. **To minimize exposure to chemotherapeutic agents you will follow these except:**

    A. Wash hands before and after handling drugs.
    B. Do not eat, drink, or store food near the administration area.
    C. Keep spill kit near administration area.
    D. Wear just gloves and a gown for preparation chemotherapy.

- Please check with a (√) to answer the following questions.

NO	Questions	True	false	I don’t know
**4.**	All types of gloves provide the same level of protection when handling cytotoxic drugs.		F	
**5.**	Labeling the chemotherapy after preparation is not important.		F	
**6.**	Safe handling of chemotherapy drugs is important because it protects healthcare workers from hazardous drug exposure.	T		
**7.**	Chemotherapy can enter the body through inhalation.	T		
**8.**	Chemotherapy can more easily enter the body through damaged skin.	T		
**9.**	Chemotherapy tablets can be chewed, cut, or crush.		F	
**10.**	Administering/handling chemotherapy is no different from administering/ handling intravenous antibiotics.		F	

Section 4

Knowledge regarding disposal of chemotherapeutic waste and managing side effects:

1. **Cytotoxic contaminated waste must be discarded in a hospital waste container with a foot pedal because:**
   A. The pedal prevents back strain.
   B. The pedal prevents the undetected spread of cytotoxic medication.
   C. This way the gloves do not get contaminated.
   D. The container opens faster.

2. **To manage spillage of chemotherapy drugs you do:**

   A. leave the spillage unattended**.**
   B. clean it after being dry
   C. instruct the cleaner to clean it
   D. put a sign at the entrance area and restrict entry.

3. **In the management of extravasation firstly you do:**

   A. remove the cannula
   B. apply a warm compress.
   C. Stop the infusion
   D. Administer antidote.

4. **Allergic to chemotherapy happened the first action you do:**

   A. Notify physician**.**
   B. Stop infusion.
   C. Administer antihistamine.
   D. Give the patient oxygen.

5. **The common side effects of chemotherapy may include:**

   A. Hair loss
   B. Nausea and vomiting
   C. Infection
   D. All of the above

6. **A disposal safety gown for handing cytotoxic drugs can be re-worn?**

   A. Yes
   B. NO

7. **All chemotherapy drugs waste should be separated from general waste.**

   A. Yes
   B. NO

8. **Educating the patient about chemotherapy drugs and it is side effects, is one of the nurse's responsibilities.**

   A. Yes
   B. NO

SECTION SCORING:

Section 2: Sum of correct answers _______/8

Section 3: Sum of correct answers _______/10

Section 4: Sum of correct answers _______/8

TOTAL KNOWLEDGE SCORING:

Sum knowledge (26 items) = scores in section 2+ scores in section 3+ scores in section 4

Scoring catagories:

1. Knowledge below 50% of the total score is considered as poor knowledge.
2. Knowledge above 50% of the total score is considered as fair knowledge.
3. Knowledge below 80% of the total score is considered as excellent knowledge.
